# Ketamine Induces Lasting Antidepressant Effects by Modulating the NMDAR/CaMKII-Mediated Synaptic Plasticity of the Hippocampal Dentate Gyrus in Depressive Stroke Model

**DOI:** 10.1155/2021/6635084

**Published:** 2021-04-23

**Authors:** Idriss Ali Abdoulaye, Shan-shan Wu, Enkhmurun Chibaatar, Da-fan Yu, Kai Le, Xue-jin Cao, Yi-jing Guo

**Affiliations:** ^1^Department of Neurology Affiliated Zhongda Hospital of Southeast University, School of Medicine, Southeast University, Nanjing, Jiangsu Province 210009, China; ^2^The Key Laboratory of Developmental Genes and Human Disease, School of Medicine, Southeast University, Nanjing, Jiangsu Province 210009, China

## Abstract

**Background:**

Ketamine has been shown to possess lasting antidepressant properties. However, studies of the mechanisms involved in its effects on poststroke depression are nonexistent.

**Methods:**

To investigate these mechanisms, Sprague-Dawley rats were treated with a single local dose of ketamine after middle cerebral artery occlusion and chronic unpredicted mild stress. The effects on the hippocampal dentate gyrus were analyzed through assessment of the N-methyl-D-aspartate receptor/calcium/calmodulin-dependent protein kinase II (NMDAR/CaMKII) pathway, synaptic plasticity, and behavioral tests.

**Results:**

Ketamine administration rapidly exerted significant and lasting improvements of depressive symptoms. The biochemical analysis showed rapid, selective upregulation and downregulation of the NMDAR2-*β* and NMDAR2-*α* subtypes as well as their downstream signaling proteins *β*-CaMKII and *α*-phosphorylation in the dentate gyrus, respectively. Furthermore, the colocalization analysis indicated a significant and selectively increased conjunction of *β*-CaMKII and postsynaptic density protein 95 (PSD95) coupled with a notable decrease in NMDAR2-*β* association with PSD95 after ketamine treatment. These changes translated into significant and extended synaptic plasticity in the dentate gyrus.

**Conclusions:**

These findings not only suggest that ketamine represents a viable candidate for the treatment of poststroke depression but also that ketamine's lasting antidepressant effects might be achieved through modulation of NMDAR/CaMKII-induced synaptic plasticity in key brain regions.

## 1. Introduction

Depression is a common psychiatric complication among ischemic stroke patients and a significant indicator of poor outcomes and a higher mortality rate [1]. Although there are variations in the reported prevalence of poststroke depression among studies, it is well accepted that it affects approximately 30% of stroke patients [2]. Even so, poststroke depression remains a poorly understood and underdiagnosed condition due to its multifactorial nature, the complexity of its pathogenesis, and the lack of a global approach to its management [3, 4]. Up to now, the standard treatment for poststroke depression in a clinical setting has been mainly pharmacological and modeled on that of depression [5]. However, such a strategy is limited due to possible differences in pathophysiology and a lack of a more integrated therapeutic approach [4].

Previous researches on the pathophysiological mechanisms of depression have pointed out the importance of glutamate in the process [6]. Extensive evidence has shown a possible link between abnormalities of the glutamate system and neuronal plasticity as well as plasticity in depressed subjects. This is even more apparent in key brain regions such as the hippocampal dentate gyrus due to its regulatory role in such processes. Indeed, studies have revealed that the dentate gyrus actively participates in the modulation of stress responses through its central role in adult neural plasticity [7, 8]. Our previous work on models of poststroke depression has shown that rodents subjected to stress had a significantly impaired dentate gyrus-related synaptic plasticity as well as glutamate circulation [9, 10]. Research on the pathophysiology of depression have not only indicated significant upregulation of the N-methyl-D-aspartate receptor- (NMDAR-) dependent calcium/calmodulin-dependent protein kinase II beta (*β*-CaMKII or CaMK2B) in the lateral habenular nucleus of the central nervous system of depressive-like animal models but also shown that some antidepressants could reduce the level of CaMK2B in the hippocampus [11]. The CaMKII family is comprised of the *α* and *β* subtypes and is not only a downstream signal molecule of NMDAR but also a second messenger of NMDAR. NMDAR-dependent calcium influx leads to reversible translocation of CaMKII from actin filament to the postsynaptic density area and initiates a bond between CaMKII and NMDAR2-*β* (NR2B), a subunit of NMDAR, therefore prolonging the CaMKII kinase activity and increasing synaptic connection [12]. NMDAR can also exert a negative effect on the formation of the spinous process by causing the contraction and disintegration of neuronal spinous processes after a long-term increase of intracellular calcium concentration [13].

Interestingly, the quest for new drugs for the treatment of major depressive disorder and other psychiatric conditions have shown that ketamine, an NMDAR antagonist, can improve patients' outcome [14–17]. A single dose of the compound has been shown to elicit favorable effects that last up to a week with minimal adverse side effects [18–20]. Investigations into its mechanisms have shown that ketamine not only inhibits NMDAR activities but also regulates the phosphorylation of *α*-CaMKII (CaMK2A) at the thr286 site in hippocampal dentate gyrus (DG) neurons of depressive-like as well as normal rodents [21–23]. Due to its rapid action and its short half-life, its lasting antidepressant effects are likely mediated by changes in synaptic-related proteins, synaptic plasticity, and/or synaptic plasticity deriving from NMDAR blockage rather than a direct impact [24, 25]. However, the fact that ketamine's action on the NMDAR complex can lead to an increase in synaptic activity and postsynaptic signaling remains a significant issue that needs clarifying. The rationale for this work derived from the absence of studies investigating the mechanisms of action of ketamine on poststroke depression and its connection with the NMDAR/CaMKII pathway as well as synaptic plasticity.

Therefore, in this study, we explored the efficacy of ketamine as an antidepressant on middle cerebral artery occlusion **(**MCAO) models with depressive-like symptoms, its impact on the NMDAR/CaMKII pathway, whether it elicits plasticity of the dentate gyrus region as well as the overall connection with its lasting effects. We hypothesized that chronic stress leads to dysregulation of the NMDAR/CaMKII pathway and synaptic plasticity in the hippocampal dentate gyrus and that ketamine not only remedies such changes but also leads to lasting antidepressant effects attained through NMDAR/CaMKII-mediated upregulation of synaptic plasticity. In this study, a single dose of ketamine was administered on Sprague-Dawley rats after middle cerebral artery occlusion and chronic unpredicted mild stress. Our study provides new insights into understanding the antidepressant mechanisms of ketamine in poststroke depression.

## 2. Materials and Methods

### 2.1. Animal Model and Experimental Design

**Figure 1:**
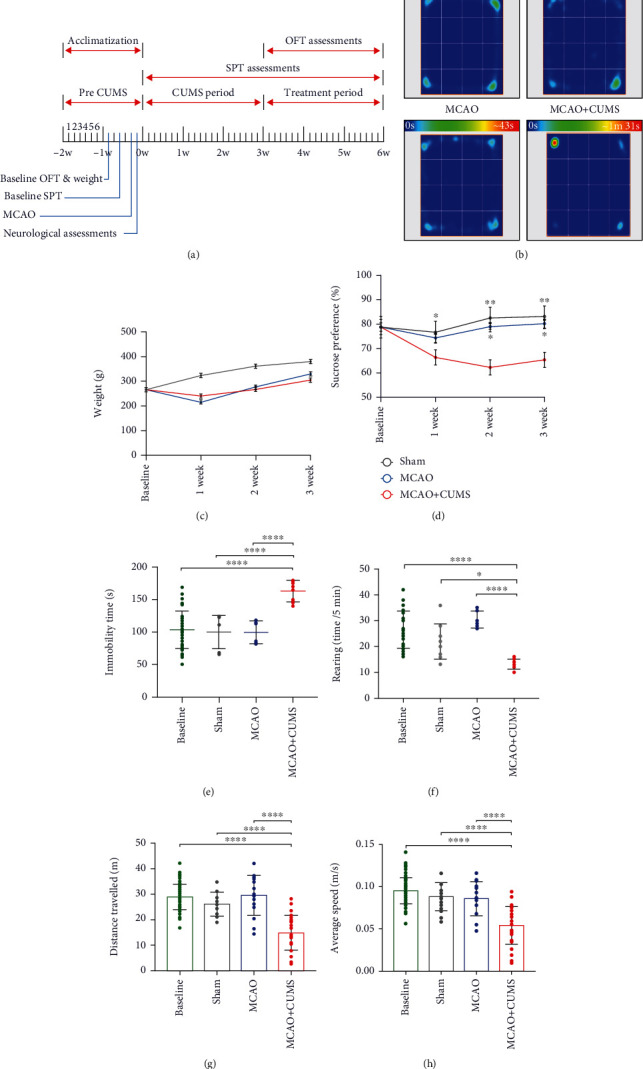
Effects of CUMS on the behaviors of MCAO rats. (a) The study schedule and behavioral testing time frames. (b) Representative heat maps of the immobility duration of each group in the OFT. (c) Dynamic variations in the weight of experimental subjects. (d) Changes in sucrose preference after a three-week stress period. (e) Pretreatment differences in immobility between groups. (f) Rearing counts of each group in the OFT. (g) Variations in distance travelled after chronic stress. (h) Changes in average speed of subjects in various groups after a three-week stress period. One-way ANOVA followed by Tukey's multiple comparison tests and repeated measures ANOVA were used for analysis. *n* = 21 per group. ^∗^*p* < 0.05, ^∗∗^*p* < 0.005, ^∗∗∗^*p* < 0.0005, and ^∗∗∗∗^*p* < 0.0001.

Adult male Sprague-Dawley (SD) rats of similar growth (230-260 g) were supplied by the medical school of Southeast University (Nanjing, China). The subjects were trained and acclimatized for 14 days in a temperature-, light-, and humidity-controlled environment (26°C, 12/12 hours light/dark, and 60%, respectively). The training was comprised of three sucrose preference tests (SPT) during which animals were provided with two bottles (water and 1% sucrose solution) for a duration of 8 hours. The bottle positions were switched after 4 hours, and the last training session values were used to establish a baseline. Additionally, open field tests were performed to establish baseline values after eight days of acclimatization and before middle cerebral artery occlusion and chronic unpredictable mild stress (Figure 1(a)).

After acclimatization and training, the SD rats (250-280 g) were divided into 4 experimental groups: the MCAO, MCAO+CUMS, MCAO+CUMS+ket, and sham groups. All except the sham group were subjected to middle cerebral artery occlusion (MCAO). The MCAO+CUMS and MCAO+CUMS+ket groups were also subjected to chronic unpredictable mild stress (CUMS), while the MCAO+CUMS+ket group received additional ketamine (ket) treatment.

All experiments were conducted during the light phase (8 AM-6 PM). All performed procedures were preapproved by the Animal Ethical and Welfare Committee of Southeast University (No. 201902150001) and complied with the National Institutes of Health Guidelines for the Care and Use of Laboratory Animals.

### 2.2. Administration of Treatment

#### 2.2.1. Middle Cerebral Artery Occlusion (MCAO)

The subjects were intraperitoneally anesthetized with sodium pentobarbital (40 mg/kg), and a midline surgical incision was performed to expose the left common carotid artery (CCA), followed by identification and isolation of the left external and internal carotid arteries (ECA and ICA). A tiny incision was made on the left CCA close to its bifurcation; then, a 3/0-gauge monofilament nylon suture coated in poly-L-lysine was guided to the origin of the left middle cerebral artery (MCA) through the ICA. The monofilament was then fixed in place and the incision sutured.

The control group also underwent a similar procedure except for incision of the CCA and insertion of a monofilament. The subject's body temperature was monitored and maintained at 37°C during the surgery. Note that the procedure is highly invasive, with an overall success rate of 80% and a survival rate of 60%. The success of the procedure was evaluated 24 hours postsurgery using the method described by Longa et al. The findings were scored using a 5-point scale (see Supplemental file (available here)), and only those with scores of 1 and 2 were retained.

#### 2.2.2. Chronic Unpredictable Mild Stress (CUMS) Model

The MCAO+CUMS and MCAO+CUMS+ket groups received a combination of 9 different stressors for a period of 3 weeks. The stressors were implemented randomly during the day or night, depending on their requirements (see Supplemental Table S1). Subjects were housed in separate cages (in different rooms) and had no contact with other rats or their stressed counterparts except when the procedure required it. Weighing and other sucrose preference tests were performed weekly to assess the overall evolution and health.

All subjects underwent behavioral tests during and at the end of the three-week stress period. Whether or not a subject is retained and included in the study depended on a comprehensive assessment of their scores and evaluation of the success of the CUMS model. Only models showing signs of depression defined by changes in behaviors on at least two of the assessed parameters (the SPT test and the immobility time or rearing test) were retained for further study. The screening method was adopted from Li et al.'s study on depressed models [26]. The model had an overall 40% success rate.

#### 2.2.3. Ketamine Administration

Ketamine hydrochloride (ket) (2 ml : 0.1 g) was obtained from the medical school of Southeast University (Nanjing, China). SD rats were divided into three different groups of 12 each (CUMS, CUMS+ket1, and CUMS+ket2) and were subjected to 3 weeks of CUMS procedures. After which, those in the CUMS+ket1 and CUMS+ket2 received a one-time dose of 1 *μ*l of ketamine at a concentration of 25 *μ*g/*μ*l, administered bilaterally and unilaterally (left side) within the dentate gyrus region using a stereotaxic frame (RWD, Shenzhen, China). The administered concentration was adapted from a previous study performed by Yang et al. [27]. OFT and SPT were performed 4 hours after ketamine treatment to evaluate and compare the viability of the two delivery methods, the antidepressant effects and subsequent changes in behaviors. The results showed that both methods yielded promising results as evidenced by improvements of behaviors in the immobility time and sucrose preference outcomes (Figures S1A and S1B). The unilateral ketamine administration method (single dose within the left hippocampal DG region) was eventually adopted for all the remaining experiments due to two reasons: (1) the bilateral injection did not allow for a long-term assessment and observation due to a very low 3-week survival rate and the requirement to significantly increase the sample size to account for mortality and (2) the two delivery methods yielded similar results, with the single administration being far less invasive to the study subjects, even though it might raise the question of hemispheric variations. All rats in the MCAO+CUMS+ket group received a single infusion of ketamine immediately after assessments of the successfulness of the CUMS procedure. The rat brain in stereotaxic coordinates was used as a reference for the acquisition of stereotaxic coordinates with minor modifications accounting for the weight difference among subjects [28]. We proceeded to verify the accuracy of the method of coordinate calculation described in the study performed by Pengfei Yang et al. [29] and utilized it to account for weight variations among our subjects. The study subjects were then evaluated by the open field test (OFT) and the sucrose preference test (SPT) depending on their respective time point. Rats in each group were sacrificed according to the established study timeframes and after testing (see Figure 1(a) for a detailed study design).

### 2.3. Biochemistry and Test Analytics

#### 2.3.1. Western Blotting, qRT-PCR, Immunofluorescence, and Transmission Electron Microscopy

Protein levels and mRNA expressions in the left hippocampal DG of the experimental subjects were measured by Western blotting and qRT-PCR using standard protocols. Meanwhile, immunofluorescence and transmission electron microscopy were used to assess the relationship between critical proteins as well as the morphological and structural changes of the area of interest. Detailed analysis methods and procedures, including Western blotting, qRT-PCR, immunofluorescence, and transmission electron microscopy are shown in the supplemental file (available here).

#### 2.3.2. Confocal Analysis

Rats were perfused with 4% paraformaldehyde before dissection and harvesting of the brain. The extracted organ was dehydrated, fixed, and sectioned into slices at -20°C using a cryostat (Leica CM1950). Coronal tissue slices from different groups were then permeabilized, blocked, and incubated overnight with Anti-CaMKII*β* (WB: 1 : 1000, IF: 1 : 100; Proteintech), Anti-CaMKII*α* (WB: 1 : 1000, IF: 1 : 100; Abcam), Anti-NMDAR2B (WB: 1 : 1000, IF: 1 : 200; Abcam), Anti-NMDAR2A (WB: 1 : 1000, IF: 1 : 100; Novus biological or Abcam), or Anti-PSD95 (WB: 1 : 1000, IF: 1 : 100; Proteintech or Abcam) at 4°C. Next, the samples were washed and incubated for 1 hour in the dark with goat anti-rabbit IgG H&L (Alexa Fluor 647) (1 : 200; Sparkjade, China), donkey anti-mouse IgG H&L (Alexa Fluor 488) (1 : 200; Abcam), or donkey anti-goat IgG H&L (Alexa Fluor 405) (1 : 200; Abcam) before mounting and visualization. Triple-stained images (Anti-CaMKII*β*/Anti-NMDAR2B/Anti-PSD95, Anti-CaMKII*α*/Anti-NMDAR2B/Anti-PSD95, Anti-CaMKII*α*/Anti-NMDAR2A/Anti-PSD95, and Anti-CaMKII*β*/Anti-NMDAR2A/Anti-PSD95) were obtained using a high-resolution laser confocal microscope (Olympus, Japan) and saved in TIFF format to avoid losses. The obtained images were then processed using the free software *ImageJ Fiji* (https://fiji.sc) and Pearson's correlation coefficient. The *coloc2* plugin of *ImageJ Fiji* which calculates several colocalization-pixel-intensity correlation-based parameters, including the Pearson's coefficient, was used to obtain the analyzed data [30]. All channels were equalized to the intensity range to avoid differences. Correlation measurements, as well as scatter diagrams, were recorded and used for colocalized fluorescence quantification and visualization. 10 sight fields in each group were analyzed.

#### 2.3.3. Sucrose Preference Test (SPT) and Open Field Test (OFT)

Rats were subjected to SPT to evaluate anhedonia during the course of the experiment. To obtain more accurate results and eliminate extreme thirst-induced bias, we decided to avoid water depriving the study subjects before testing. As a result, the testing duration was extended to 8 hours. Water and sucrose solution (1%) intakes were assessed by weighing the two bottled solutions before and after testing. The two bottle positions were randomly assigned (left/right side of the cage) and carefully switched at the half time point (4 hours). During SPT, the usually pair-housed animals in the MCAO and sham groups were separated and single-housed for the duration of the testing. The baseline value was obtained under similar conditions before any surgical or stress procedure, and SPT was performed weekly during the course of the study. The sucrose consumption value was calculated using the following formula:

sucrose preference (%) = (sucrose intake (g)∗100)/(sucrose intake (g) + water intake (g)).

Locomotion, immobility duration, and rearing were evaluated using the OFT. The testing room was ventilated, and the testing apparatus sterilized 48 hours before testing. Meanwhile, animals were left to acclimate to the testing environment for a day. During testing, each subject was placed at the center of the open field, consisting of a black floored 75 cm long × 75 cm wide × 40 cm high wooden box. The floor was divided into 25 equal squares, each 1 cm wide. The tests were performed in a quiet and dimly lit room with adequate and balanced illumination of the testing box. Behaviors were recorded during a period of 5 minutes. The OFT was performed by experienced technicians. Scoring and analyses were carried out automatically by the ANY-maze Behavior Tracking Software (Stoelting Co., USA).

### 2.4. Statistical Analysis

Statistical analyses were performed, and data plotted on SPSS Version 22 (IBM Corp., NY) or PRISM 8.0 (GraphPad Software, Ca). The quantitative data of Western blot and real-time quantitative PCR are relative magnitudes that were normalized with *β*-actin protein and mRNA expressions. Data were expressed as mean ± standard deviation (SD). SPT and weight data were analyzed using repeated-measures one-way ANOVA. Differences in OFT, protein expressions, and other key factors among multiple groups were assessed by one-way analysis of variance (Tukey). *Fiji* software and Pearson's correlation coefficients were used to calculate fluorescence correlation coefficients for the colocalization analysis [31]. *p* values < 0.05 were considered statistically significant.

## 3. Results

### 3.1. CUMS Protocol Induces Depressive-Like Behaviors in MCAO Models

To evaluate the impact of stress on MCAO rats, subjects in each group underwent a series of behavioral tests during the stress period and after three weeks of stress regimen. In this study, the OFT results demonstrated that the investigative abilities of the stressed subjects were decreased significantly. Overwhelmingly, the MCAO+CUMS group did not only exhibited a significant weight loss due to stress and anorexia (Figure 1(c)) but also a gradual increase in anhedonia degree after the initial week of stress (Figure 1(d)). As shown in Figures 1(b), 1(e)–1(h), significant changes in behaviors defined by a higher heat signature, longer immobility periods, a decrease in rearing behavior, and significant reduction in time travelled and speed observed in the stressed subjects compared to the sham and MCAO groups (*p* < 0.05, *n* = 21 per group and time point) which indicated depressive-like symptoms. These results further confirm the validity of the poststroke model.

### 3.2. Ketamine Administration Alleviates the Depressive-like Symptoms in MCAO+CUMS Rats

**Figure 2:**
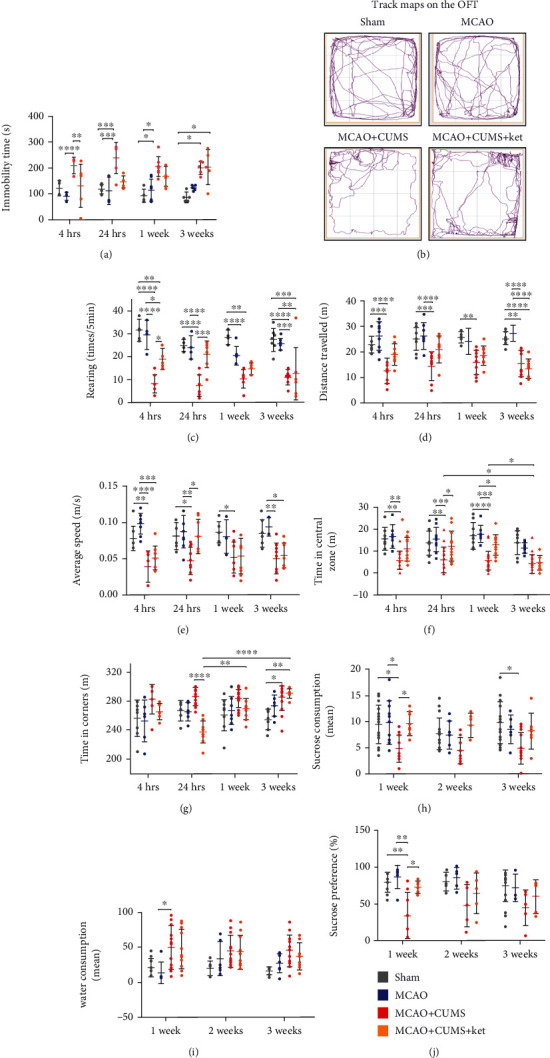
Effects of ketamine on the behaviors of depressive-like MCAO rats. (a) Comparison of the immobility duration of the study groups after ketamine administration (OFT). (b) Representative movement tracks of different groups (OFT). (c) Rearing counts of various groups after ketamine treatment. (d) Changes in distance travelled after ketamine treatment. (e) Temporal improvements in average speed after ketamine administration. (f) Variations in explorative time spent in the central zone of each group. (g) Variations in time spent in the corner zone after ketamine treatment. (h) Changes in sucrose consumption after ketamine administration (SPT). (i) Differences in water consumption between groups at various time points. (j) Temporal variations in sucrose intake preference after ketamine treatment. 1w: 1 week. One-way ANOVA followed by Tukey's multiple comparison tests. *n* = 16 per group. ^∗^*p* < 0.05, ^∗∗^*p* < 0.005, ^∗∗∗^*p* < 0.0005, and ^∗∗∗∗^*p* < 0.0001.

The results showed that ketamine elicited a rapid antidepressant effect on MCAO+CUM + Ket group with significant improvements in depressive-like behaviors as evidenced by better immobility and rearing activities observed within 4 h post-treatment (Figures 2(a)–2(c)) (*p* < 0.05, *n* = 16). The OFT also showed that treated depressive subjects exhibited better explorative behaviors as indicated by better results in parameters including average speed and time spent in different zones than their nontreated counterparts (Figures 2(d)–2(g)) (*n* = 16). Additionally, the SPT also showed a significant improvement of anhedonia among the MCAO+CUM+ket group as compared to its nontreated counterpart (MCAO+CUM, *p* < 0.05, *n* = 16) (Figures 2(h)–2(j)).

### 3.3. Ketamine Administration Selectively Regulates NMDAR Subunits in the Hippocampal DG Region of MCAO+CUMS Rats

**Figure 3:**
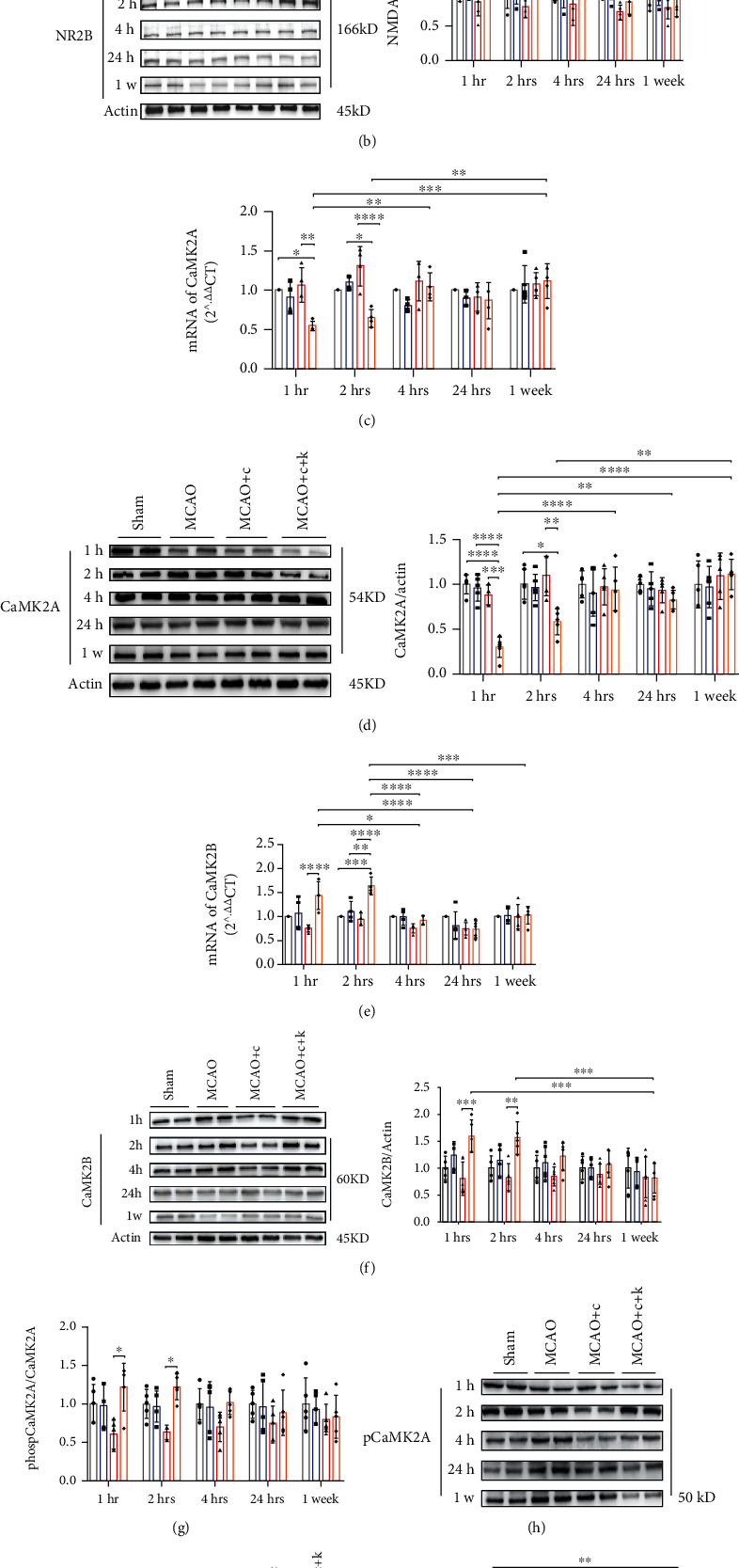
Changes in NMDAR and CaMKII subunits after ketamine administration. (a) Representative immunoblots and NMDAR2-*α* (NR2A or NMDAR2A) expressions in the hippocampal DG of various study subjects. (b) Representative immunoblots and NMDAR2-*β* (NR2B or NMDAR2B) expressions in the hippocampal DG of various groups after ketamine. (c) Relative mRNA expression of CaMK2A in the hippocampal DG region of the brain. (d) Representative immunoblots and protein expression of CaMK2A in the DG region of various test subjects. (e) Relative mRNA expression of CaMK2B in the hippocampal DG region of various study groups. (f) Representative immunoblots and protein expression of CaMK2B in the hippocampal DG region of the brain. (g) Protein levels of the phosphorylated state of CaMK2A (pCaMK2A or phospCaMK2A) in different groups. (h) Representative immunoblots of pCaMK2A expressions. (i) Representative immunoblots and protein levels of the phosphorylated state of CaMK2B (pCaMK2B or phospCaMK2B) in the GD region of various experimental subjects. 1w = 1 week. MCAO+c: MCAO+CUMS; MCAO+c+k: MCAO+CUMS+ketamine. One-way ANOVA followed by Tukey's multiple comparison tests. *n* = 6 per group. ^∗^*p* < 0 05, ^∗∗^*p* < 0.005, ^∗∗∗^*p* < 0.0005, and ^∗∗∗∗^*p* < 0.0001.

As cited above, ketamine's antidepressant properties have been closely connected with its antagonistic properties toward the NMDAR complex. Therefore, we set out to examine the impact of ketamine administration on individual NMDAR subunits. Protein quantification of NMDAR from hippocampal DG tissues showed that administration of ketamine significantly reduced the expressions of the NR2A subunit (Figures 3(a)) (*p* = 0.0411 for the 1-hour time point, and *p* = 0.0418 for the 2 hours, MCAO+CUMS+ket vs. MCAO+CUMS, *n* = 6). While concomitantly, subjects in the MCAO+CUMS group had lower NR2B subunit expressions; an effect that was remedied by ketamine administration (Figures 3(b)) (*p* = 0.0401 for the 1-hour time point and *p* = 0.0385 for the 2 hours, MCAO+CUMS+ket vs. MCAO+CUMS, *n* = 6). The results also showed that ketamine's effects on both variants were transient and lasted for less than 4 hours. These findings were further supported by the immunofluorescence results on the expressions of NR2A and B proteins (Supplemental Figures S2 and S3). Further analysis of the protein expressions of remaining NMDAR subunits, NMDAR1 (NR1) and NMDAR3 (A/B) (NR3), showed that ketamine did not have a significant effect on these variants (Supplemental Figures S1C and S1D). Taken together, these findings suggested that ketamine's antidepressant actions are selective, NR2A- and 2B-dependent, and are exerted through regulation of those specific subunits.

### 3.4. Ketamine Administration Modulates NMDAR's Downstream Signaling Pathways through Regulation of *α*- and *β*-CaMKII Expressions

Next, we proceeded to investigate the impact of ketamine's regulation of NR2A and 2B on the CaMKII family, a key player in ketamine's rapid response [24]. Analysis on CaMK2A indicated that ketamine rapidly lower its protein levels and mRNA expression in MCAO+CUMS+ket rats (Figures 3(c) and 3(d)) (*p* = 0.0002 and *p* = 0.0034, respectively; MCAO+CUMS+ket vs. MCAO+CUMS; *n* = 6), an effect that lasted for about 2 hours (*p* = 0.0042 and *p* < 0.0001, respectively; MCAO+CUMS+ket vs. MCAO+CUMS; *n* = 6). The immunoblotting and mRNA quantifications indicated that contrary to CaMK2A, CUMS downregulated CaMK2B expressions in the DG region of MCAO+CUMS rats. A trend immediately alleviated by ketamine. Indeed, the results showed that ketamine administration elicited a significant and rapid upregulation of CaMK2B protein levels as well as mRNA expression in MCAO+CUMS+ket rats (within an hour of administration) (Figures 3(e) and 3(f)) (*p* = 0.0401 and *p* < 0.0001, respectively, MCAO+CUMS+ket vs. MCAO+CUMS, *n* = 6), an effect that lasted for around 2 hours (*p* = 0.0385 for protein levels and *p* < 0.0001 for mRNA expression, MCAO+CUMS+ket vs. MCAO+CUMS, *n* = 6). These results suggested that ketamine, through its actions on the NMDAR, rapidly regulates downstream signaling pathways accounting for its rapid response. Additionally, elevation or decrease of the CaMKII subunits is the result of changes in their transcription levels due to ketamine-regulated NMDAR expressions. The above findings were further corroborated by the immunofluorescence analysis, which indicated stronger CaMK2B and weaker CaMK2A signals in stained DG tissues of treated depressive-like MCAO rats (Supplemental Figures S2 and S3).

Subsequently, we attempted to investigate whether the above ketamine-induced changes were reflected by variations in their phosphorylated forms. The immunoblotting results showed a negative correlation between changes in CaMK2B as well as CaMK2A protein expressions and their phosphorylated versions (Figures 3(g)–3(i)). Indeed, ketamine reduced the phosphorylation of CaMK2B while increasing that of CaMK2A ((*p* = 0.0121 after 1 hour and *p* = 0.0308 after 2 hours) and (*p* = 0.0191 after 1 hour and *p* = 0.0292 after 2 hours), respectively; MCAO+CUMS+ket vs. MCAO+CUMS), an effect that was maintained up to 2 hours after administration. Together, the results suggested that ketamine might utilize a selective regulatory process on the NMDAR/CaMKII pathway to achieve its rapid antidepressant-like properties and those properties are potentially dependent on CaMK2A and 2B.

### 3.5. Ketamine Administration Induces Synaptic Plasticity in the Hippocampal DG Region of MCAO+CUMS Rats

**Figure 4:**
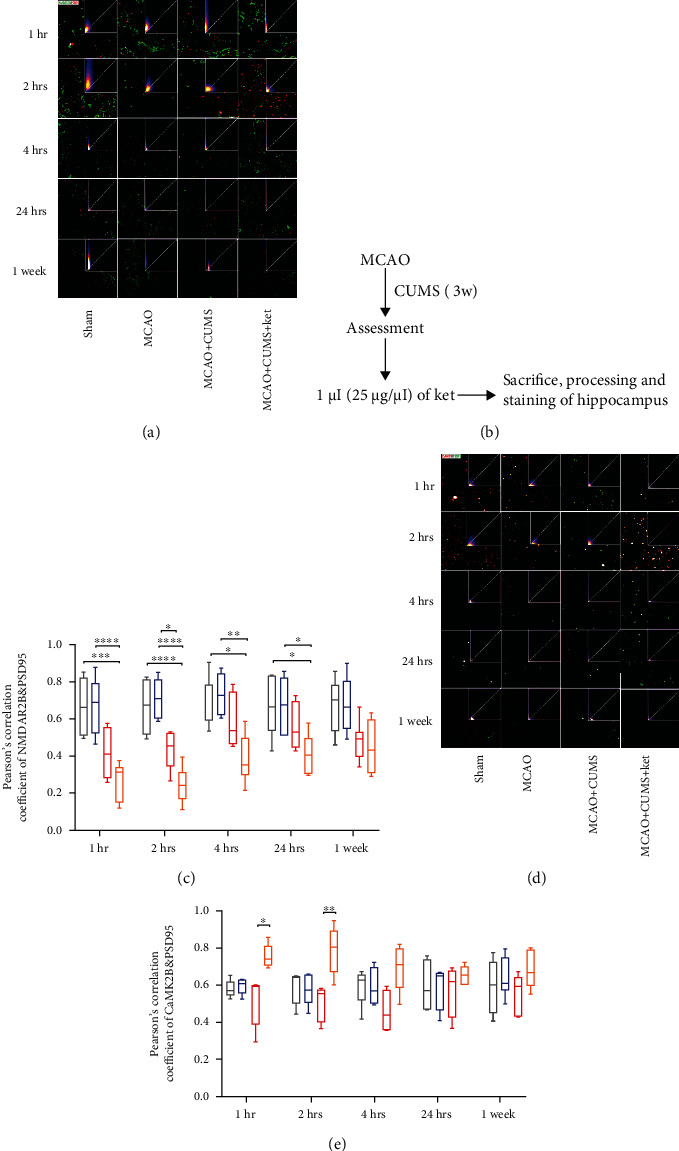
Analysis of the relationship between the NMDAR/CaMKII pathway and changes at the postsynaptic level by colocalization. (a) Representative confocal images of the relationship between NR2B (labeled in green) and PSD95 (labeled in red). The upper right corner of each image shows the corresponding scattergram with pixel concentration along the diagonal, depending on the degree of colocalization. (b) Schedule and procedures before image acquisition. (c) Colocalization between NR2B and PSD95, as analyzed by Pearson's correlation coefficients. (d) Representative confocal images of the relationship between CaMK2B (labeled in red) and PSD95 (labeled in green). The upper right corner of each image shows the corresponding scattergram with pixel concentration along the diagonal, depending on the degree of co-localization. (e) Colocalization between CaMK2B and PSD95, as analyzed by Pearson's correlation coefficients. Scale bar: 20 *μ*m. Pearson's correlation coefficient analysis was based on ten sight fields in each group. *n* = 6 per group. 1 hr: 1 hour, 2 hrs: 2 hours, 4 hrs: 4 hours, 24 hrs: 24 hours, and 3 w: 3 weeks. ^∗^*p* < 0.05, ^∗∗^*p* < 0.005, ^∗∗∗^*p* < 0.0005, and ^∗∗∗∗^*p* < 0.0001.

We utilized laser confocal microscopy and Pearson's correlation coefficient analysis to determine the colocalization of synapses (PSD95 as a marker) with CaMK2B, synapses with CaMK2A, synapses with NR2B, and synapses with NR2A (Figure 4(b)). The results indicated that CUMS reduced the correlation of PSD95 with NR2B as evidenced by decreased Pearson' correlation coefficients, an effect that was considerably exacerbated by the administration of ketamine (Figures 4(a) and 4(c)) (MCAO+CUMS+ket vs. sham, *p* = 0.0001, *p* < 0.0001, *p* = 0.0212, and *p* = 0.0486 at 1 hour, 2 hours, 4 hours, and 24 hours' time points, respectively, *n* = 6). The colocalization experiments between PSD95 and CaMK2B indicated a significant increase in colocalized puncta after ketamine administration, effects that lasted for more than 2 hours (Figures 4(d) and 4(e)) (MCAO+CUMS+ket vs. MCAO+CUMS, *p* = 0.0001, *p* < 0.0458, and *p* = 0.0061, 1 and 2 hours after administration, respectively, *n* = 6). Interestingly, analyses of colocalization between PSD95 and NR2A, as well as, CaMK2A did not show any significant variation in Pearson's correlation coefficients between groups (Supplemental Figure S4). These findings meant that ketamine influences synaptic connections and functions possibly through reversible dissociation of the NR2B/PSD95 complex and translocation of CaMK2B to postsynaptic density areas to form new CaMK2B/PSD95 complexes.

**Figure 5:**
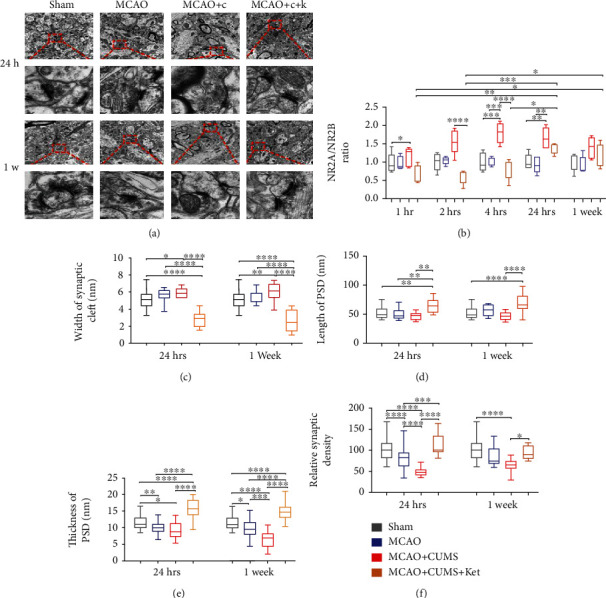
Effects of ketamine on synaptic plasticity in the hippocampal DG region of depressive-like MCAO rats. (a) Representative transmission electron microscopy images of synapses in the hippocampal DG region of various study groups at two different time points (10000x and 80000x) (*n* = 3 rats). Scale bars: 1 *μ*m and 0.1 *μ*m, respectively. (b) The NR2A/NR2B ratio between different groups at different time points. (c) Comparison of the average width of synaptic cleft after ketamine administration. (d) Comparison of the average length of postsynaptic density between groups after treatment. (e) Comparison of the thickness of postsynaptic density between various groups one day and two weeks after treatment. (f) Comparison of the relative synaptic density between groups. 1 hr: 1 hour, 2 hrs: 2 hours, 4 hrs: 4 hours, and 24 hrs: 24 hours; PSD: postsynaptic density. One-way ANOVA followed by Tukey's multiple comparison tests. *n* = 6 per group. ^∗^*p* < 0.05, ^∗∗^*p* < 0.005, ^∗∗∗^*p* < 0.0005, and ^∗∗∗∗^*p* < 0.0001.

Next, we assessed the overall structural significance of such processes in the DG region of the brain. Synaptic plasticity was evaluated through quantification and examination of ultrastructure in the hippocampal DG region. In this study, the analysis revealed that CUMS increased the NR2A/NR2B ratio for a period of up to a week and that administration of ketamine not only reversed those effects but also further lowered the ratio for more than 4 hours compared to both the sham and MCAO groups (Figure 5(b)). The ultrastructure analysis revealed that CUMS impaired synaptic plasticity as evidenced by the widening of the average distance between the pre- and postsynaptic membranes (Figures 5(a) and 5(c)) (MCAO+CUMS vs. sham, *p* = 0.0432 and *p* = 0.0031, 24 hours and 1 week, respectively, *n* = 6), the lower relative synaptic density of the MCAO+CUMS group (Figure 5(f)) (*p* < 0.0001, 24 hours and 1 week, MCAO+CUMS vs. sham; *p* < 0.0001, 24 hours, MCAO+CUMS vs. MCAO, *n* = 6), and the loss of thickness of postsynaptic density in the MCAO+CUMS group (Figure 5(e)) (*p* = 0.0443 and *p* < 0.0001, 24 hours and 1 week, MCAO+CUMS vs. sham, *n* = 6). As seen in Figures 5(c)–5(f), ketamine administration not only reversed the ultrastructural changes seen after CUMS but also showed that it promoted synaptic plasticity in the hippocampal DG region of MCAO+CUMS+ket models. Together, these findings suggested that ketamine might selectively utilize its rapid regulation of the NMDAR/CaMKII pathway to achieve significant and lasting (up to a week) synaptic plasticity in the DG region, further extending its properties.

## 4. Discussion

Depression represents a significant complication for stroke patients, further increasing the burden on caregivers as well as relatives [32]. The generated quantifiable behavioral changes are often just the manifestations of much profound and earlier alterations. Exposure to stress has been shown to have lasting and detrimental effects on the hippocampus, whether at the functional, molecular, or structural levels [33–36]. Our previous study has shown signs of dysregulation of glutamate circulation, neural regeneration, and synaptic plasticity in the DG region of depressive-like MCAO rats [9, 10], results that were further confirmed by the current study (Figure 5). Such alterations led to abnormal behaviors easily detectable in experimental subjects, as evidenced by our pretreatment results (Figure 1). Ketamine, as a noncompetitive NMDAR antagonist, has received lots of attention during the years due to its antidepressant properties [37–39]. Although the mechanisms of action of ketamine are still being explored, it is evident that a lot remains to be elucidated. In this study, the obtained data showed that administration of ketamine leads to rapid and lasting antidepressant effects in depressed stroke models. Most importantly, our results not only provide a possible link between ketamine's rapid antidepressant effects and its selective regulation of the NMDARs and their downstream CaMKII signaling in the hippocampal DG region of the brain but also show that ketamine's lasting antidepressant properties are likely to be the results of synaptic plasticity mediated by selective modulation of the NMDAR/CaMKII pathway. Therefore, this study explains ketamine's rapid and persistent antidepressant effects in poststroke depression models.

The NMDARs are essential for synaptic plasticity through their control of the postsynaptic ***α***-amino-3-hydroxy-5-methyl-4-isoxazole propionic acid receptors (AMPARs) system as well as their direct structural modulating effects [40]. Activation of the NMDAR system is a prerequisite for postsynaptic calcium influx, a key component for AMPARs trafficking, therefore synaptic plasticity [41]. Excessive glutamate in the synaptic region causes abnormal activation and modulation of the NMDAR system leading to depression-like symptoms [42, 43]. Interestingly, our results showed that the administration of ketamine selectively modulated the previously impaired NR2A and NR2B subunits in depressive-like MCAO rats while having no effects on the NR1 and NR3 variants. These results demonstrated that the effects of ketamine are not directed at all NMDARs but rather NR2A and NR2B subunits dependent. It provides further proof that ketamine's antidepressant effects and the NR2A and NR2B subunits are intrinsically linked at the functional level since the four NMDAR2 (A, B, C, and D) subunits essentially encompass the physiological role of NMDARs [44]. Previous findings have shown that NR2B localization at synaptic sites is essential for the induction of long-term potentiation (LTP) and promotes its interaction with PSD95 and the CaMKII complex, a process crucial for synaptic plasticity [45–47]. In this study, the colocalization results showed that ketamine interferes with NR2B and CaMK2B but not NR2A and CaMK2A localization at synapses by promoting the interaction of CaMK2B/PSD95 rather than NR2B/PSD95. It implied that the effects of ketamine on the NMDAR/CaMKII pathway are much more targeted and intricate. It is likely that the administration of ketamine rapidly initiated a shift in CaMK2B state from active (phosphorylated) to inactive, thus significantly increasing its availability for synaptic translocation. To our knowledge, this is the first time such specificity has been reported. The choice of NR2B upregulation by ketamine might be explained by the preferential impact of stress on glutamate circulation and the unique properties of each NMDAR subunit [48–50] since it has been previously found that specific alterations of the NMDAR subunits lead to distinct traits, such as enhanced learning and memory in cases of NR2B overexpression [40, 51]. Furthermore, the NR2s are the primary regulators of the open/close state of NMDAR, and as such, NR2A can undergo reverse calcium-dependent inactivation, a property not shared by NR2B [52]. Besides, the NR2B receptors might play a more preeminent role in the regulation of the density and strength of glutaminergic synapses [53] due to their higher sensitivity to glutamate release compared to their NR2A counterparts, which mediate a more direct synaptic transmission [54]. These findings further indicate the complexity of the molecular mechanisms of ketamine in poststroke depression conditions.

Previous works have already established the crucial role of the CaMKII family in the downstream signal transduction of NMDAR [55–57]. Evidence has highlighted the primary role played by the interaction of those two complexes in information processing and mood regulation [58]. Calcium influx resulting from NMDAR activation is a significant step for the formation of a CaMKII/NR2B complex and, by extension, synaptic connections [12, 59]. CaMKII is abundantly found at the glutaminergic postsynaptic density and is activated by Ca^2+^-calmodulin deriving from NMDAR opening, effects that last long after the stimulus has subsidized [60]. Prior studies have shown that a bond between CaMKII and the NR2B subunit is necessary for LTP induction, and disruption of the NR2B/CaMKII complex can lead to the downregulation of LTP as well as CaMK2A autophosphorylation in the hippocampus [61–63]. Additionally, CaMKII and PSD95 are essential for synaptic NR2B anchorage and stabilization [47]. The current results show that ketamine administration rapidly promoted the expression of CaMK2B in the hippocampal DG region while decreasing that of the CaMK2A variant. Interestingly, the opposite effects were observed in the quantification of their phosphorylated states, further suggesting that the selective modulation of NMDAR by ketamine elicits a specific shift from an activated state to deactivated or vice versa, depending on the CaMKII variant. Interactions between CaMKII and NMDAR subunits are essential for synaptic plasticity and learning [63], as evidenced by the current findings.

Synaptic plasticity represents the ability of the brain to make essential and dynamic adjustments to stimuli and is intrinsically linked with the pathophysiology of conditions such as depression [64, 65]. It is well documented that chronic stress generates abnormal morphological changes in key brain regions at the ultrastructural level [66, 67]. Previous studies have shown that the pathophysiological hallmarks of depression are not only the observed impair dentate gyrus neurogenesis and cell death but also a significant reduction in the number of synapses and axons, therefore, lowering network connectivity in critical regions of the brain [68, 69]. Our findings showed that stress increases the NR2A/NR2B ratio and ketamine had a rapid and significant lowering effect on that parameter. It further supports the indication that stress impairs synaptic plasticity since the balance between NR2A and NR2B is a primary indicator of experience-driven plasticity as well as a modulator of the long-term potentiation/long-term depression in the adult nervous system [40, 41, 70]. Therefore, by lowering the NR2A/NR2B ratio, ketamine induces massive ultrastructural changes at the synaptic level. These findings are further substantiated by results depicted in Figure 5 indicating that MCAO rats subjected to the CUMS protocol underwent significant detrimental restructuring and morphological changes in their hippocampal DG region and that the administration of ketamine not only remedied such effects but also actively promoted the increase of synaptic density and connectivity, an effect that lasted for several days parallel to its antidepressant properties.

There are limitations worth noticing in this study. Firstly, we did not perform a genetic knockout of individual components of the NMDAR/CaMKII pathway to definitively establish the role of the pathway in ketamine's antidepressant effects. Secondly, we did not investigate the mechanism by which ketamine can differentiate and selectively target the phosphorylation of these two CaMKII subunits and whether or not the remaining variants also play a role in its effects. Thirdly, the antidepressant mechanism of ketamine is multitargeted and involves a synergistic interaction between several pathways, and as such, whether other known ketamine-related pathways play a crucial role in the resultant synaptic changes needs further investigation.

**Figure 6:**
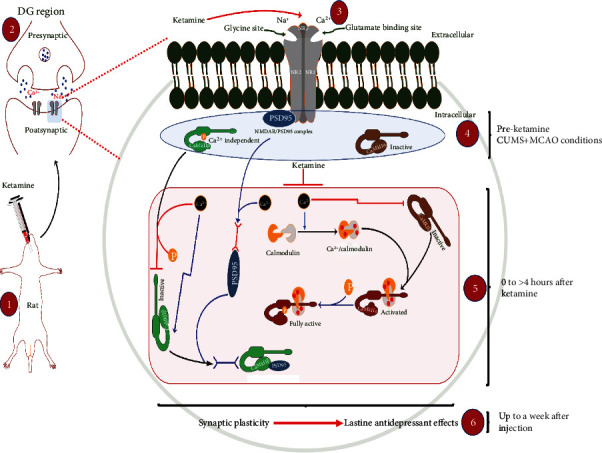
Ketamine-mediated lasting antidepressant effects result from NMDAR/CaMKII induced synaptic plasticity. A proposed model for the rapid and lasting actions of ketamine through the NMDAR/CaMKII pathway is presented. Administration of ketamine regulates calcium influx through its action on NMDAR resulting in rapid inhibition of CaMK2B phosphorylation and transcriptional stimulation of its inactive form. At the same time, ketamine promotes the dissociation of PSD95 from the NR2B/PSD95 complex in favor of newly formed CaMK2B/PSD95 complexes, leading to a significant stimulation of synaptic plasticity. In parallel, ketamine's regulation of intracellular calcium levels also leads to rapid phosphorylation of CaMK2A, further supporting the plasticity process through stimulation of plasticity-related proteins (PSD95). Overall, the results suggest that ketamine's temporary regulation of the NMDAR/CaMKII pathway not only accounts for its rapid actions but also initiates profound and lasting changes (<a week) at the postsynaptic level leading to its lasting antidepressant effects. The illustration was partially based on motifs from http://motifolio.com.

In conclusion, ketamine represents a promising candidate for the treatment of depression among stroke patients and perhaps more studies should be directed toward understanding its mechanism. In this case, our data shows that ketamine's rapid antidepressant actions are probably mediated through the NMDAR/CaMKII pathway and result in a significant increase in synaptic plasticity in the DG region of depressive-like models of stroke (Figure 6), making it a viable option for the treatment of depression in stroke patients, and linking its lasting antidepressant properties with the resulting synaptic plasticity in key brain regions.

## Data Availability

The datasets used and analyzed during the current study are available from the corresponding author on reasonable request.

## Supplementary Materials

Supplementary Materials The supplementary material contain additional supportive data, table, and figures cited in this article such as a detailed CUMS protocol.
