# Supplementary material for: Ketamine Induces Lasting Antidepressant Effects by Modulating the NMDAR/CaMKII-Mediated Synaptic Plasticity of the Hippocampal Dentate Gyrus in Depressive Stroke Model
Source: Neural Plast. 2021 Apr 23;2021:6635084. doi: 10.1155/2021/6635084 (PMC8088363; doi:10.1155/2021/6635084)
Supplement: Supplementary Materials — The supplementary material contain additional supportive data, table, and figures cited in this article such as a detailed CUMS protocol. [file 6635084.f1.docx]

**Ketamine induces lasting antidepressant effects by modulating the NMDAR/CaMKII mediated synaptic plasticity of the hippocampal dentate gyrus in depressive stroke model**

Abdoulaye Idriss Ali^1^, Shan-shan Wu^1^, Enkhmurun Chibaatar^1^, Da-fan Yu^1^, Kai Le^1^, Xue-jin Cao^1^, Yi-jing Guo^1,2*^

^1^Department of Neurology Affiliated Zhongda Hospital of Southeast University, School of Medicine, Southeast University, Nanjing, Jiangsu Province, 210009, China

^2^The Key Laboratory of Developmental Genes and Human Disease, School of Medicine, Southeast University, Nanjing, Jiangsu Province, 210009, China

*Correspondence should be addressed to Yi-jing Guo − Department of Neurology, Affiliated Zhongda Hospital of Southeast University, Nanjing, Jiangsu Province 210009, China; orcid.org/0000-0002-6638-8963; Phone: 0086-25-83262241; Email: 101010270@seu.edu.cn.

**Supplementary Materials**

**Supplemental Methods and Materials**

**Reagents and Antibodies**

Ketamine hydrochloride (2ml:0.1g) was procured from the medical school of the Southeast University (Nanjing, China), diluted with 0.9% NaCl (saline) to obtain a concentration of 25 μ g/μ l, and stored at 4°C for later use. The used primary antibodies were: Anti-CaMKIIβ (WB: 1:1000, IF: 1:100; Proteintech), Anti-CaMKIIα (WB: 1:1000, IF: 1:100; Abcam), Anti-Phospho-CaMKII (Thr286) (1:1000; Cell Signaling Technology), Anti-NMDAR2B (WB: 1:1000, IF: 1:200; Abcam), Anti-NMDAR2A (WB: 1:1000, IF: 1:100; Novus biological or Abcam), Anti-NMDAR1 (WB:1:1000; Abcam), Anti-NMDAR3A/NMDAR3B (1:1000; ThermoFisher Scientific), Anti-PSD95 (WB: 1:1000, IF: 1:100; Proteintech or Abcam), and β-Actin (1:3000; Proteintech or Abcam). Used secondary antibodies were: HRP Affinipure goat anti-rabbit IgG (H+L) (1:3000; Biosharp), HRP Affinipure goat anti-mouse IgG (H+L) (1:3000; Biosharp), goat anti-rabbit IgG H&L (Alexa Fluor 647) (1:100; Sparkjade, China), donkey anti-mouse IgG H&L (Alexa Fluor 488) (1:100; Abcam), and donkey anti-goat IgG H&L (Alexa Fluor 405) (1:100; Abcam).

**Post-surgical assessment and scoring:**

The scoring standard was established as follows: a 0 score indicates no neurologic deficit, a score of 1 (failure to fully extend the right forepaw) indicates a mild focal neurologic deficit, a score of 2 (circling to the right) indicates a moderate focal neurologic deficit, and a score of 3 (falling to the right) indicates a severe focal deficit; rats with a score of 4 failed to walk spontaneously and had a depressed level of consciousness(Longa, Weinstein et al. 1989).

**CUMS protocol and schedule**

**Table S1. the daily administered stress schedule, duration, and type**

|  | **Monday** | **Tuesday** | **Wednesday** | **Thursday** | **Friday** | **Saturday** | **Sunday** |
| --- | --- | --- | --- | --- | --- | --- | --- |
| **First week** | 10 a.m swimming in water at 4°C for 5 min |  | 9 a.m end of water deprivation  9 a.m-10 a.m exposure to empty bottle  10 a.m water resumption |  | 9 a.m end of tilted cage | 7 a.m end of overnight lighting  10 a.m body weighing | 9 a.m end of soiled cage  10 a.m-6 p.m SPT |
|  |  | 4 p.m-9 a.m water deprivation | 7 p.m-9 p.m exposure to stroboscopic lightning | 4 p.m-9 a.m tilted cage | 7 p.m-7 a.m continuous lighting | 4 p.m-9 a.m soiled cage | 7 p.m foot shock |
| **Second week** | 10 a.m-12 a.m paired housing | 9 a.m foot shock | 10 a.m end of water and food depravation | 10 a.m swimming in water at 4°C for 5 min |  | 10 a.m body weighing | 9 a.m end of tilted cage  10 a.m-6 p.m SPT |
|  |  | 2 p.m-10 a.m water and food depravation | 3 p.m tail pinch |  | 7 p.m-9 p.m exposure to stroboscopic lightning | 4 p.m-9 a.m tilted cage |  |
| **Third week** |  | 9 a.m end of water deprivation  9 a.m-10 a.m exposure to empty bottle  10 a.m water resumption |  | 9 a.m end of tilted cage | 9 a.m foot shock | 7 a.m end of overnight lighting  10 a.m body weighing | 9 a.m end of soiled cage  10 a.m-6 p.m SPT |
|  | 4 p.m-9 a.m water deprivation | 3 p.m tail pinch | 4 p.m-9 a.m tilted cage | 2 p.m-4 p.m paired housing | 7 p.m-7 a.m continuous lighting | 4 p.m-9 a.m soiled cage |  |

**Western blotting**

**Sample preparation:** Rats were sacrificed after behavioral tests. Brains were retrieved, and the two hemispheres were quickly separated before snap freezing. The left hemisphere was then sectioned on a Leica CM1950 cryostat where the hippocampal dentate gyrus tissues were isolated, dissected, and stored at -80 °C for later use. Tissues were homogenized and total protein extracted using a Radioimmunoprecipitation assay **buff**er (RIPA lysis buffer) (Beyotime)+**phenylmethylsulfonyl fluorid**e (PMSF) (100mM) (Beyotime). The samples were then left for 2 hours on an orbital shaker at 4°C before centrifugation (12000 rpm for 20 min at 4°C). The subsequent supernatants were transferred into new Eppendorf tubes, and the protein concentration was measured using a **bicinchoninic ac**id (BCA) protein quantification assay (Beyotime or KeyGEN BioTECH) per the manufacturer instructions. Equal volumes of 2x-SDS PAGE sample loading buffer (Beyotime) were added to the samples before denaturing (boiling at 100°C for 5min).

**Loading and running:** Equal amounts of protein (from the previously prepared samples) as well as a molecular weight marker (ThermoFisher Scientific) were loaded into SDS-PAGE gel (Beyotime) wells and ran for approximately 2-3 hours at 100 volts.

**Transferring and staining:** **Polyvinylidene fluorid**e (PVDF) membranes (Merck Millipore) were activated with methanol before transfer initiation. After the transfer, membranes were blocked for 1 hour at room temperature using a 5% non-fat milk in **Tris-buffered saline with 0.1% Tween** (TBST) solution. The membranes were then incubated overnight at 4°C with corresponding primary antibodies in the blocking solution. After three washes (5 min each) with TBST, the membranes were incubated with the corresponding secondary antibody for 1 hour at room temperature. A chemiluminescent reagent ECL (Biosharp) was used for signal development after three washes (5 min each) with TBST. Image acquisition was performed on ImageQuant LAS 4000 (GE).

**qRT-PCR Analysis**

CaMK2B and 2A mRNA expressions were measured 1 hour, 2 hours, 4 hours, one day, and one week after ketamine administration. Total mRNA of the left hippocampal DG was extracted by Trizol (ThermoFisher Scientific, USA) per the manufacturer’s instructions. After reverse transcription into cDNA (RevertAid first-strand cDNA synthesis kit (ThermoFisher Scientific, USA)), the samples were amplified with SYBR® green PCR master mix (ThermoFisher Scientific, USA). The StepOneTM Software 2.3 (ThermoFisher Scientific, USA) was used for quantification. The used primer sequences were as follows: CaMKIIα (228 bp), forward primer 5'- CCTTCACGCCATCATTCTT-3', reverse primer 5'-CCATCAACCCGTCCAAAC-3' and CaMKIIβ (193 bp), forward primer 5'-GGGATGGGAAGTCATAGGC-3', reverse primer 5'- TTTGGATTTGCGGGAACA-3'. The expressions were calculated as 2^-ΔΔCt^.

## Immunofluorescence assay

Rats were perfused with 4% paraformaldehyde before harvesting. After harvesting, the brain tissues were left to dehydrate in a 30% sucrose solution. Following dehydration, the tissues underwent fixation with 4% paraformaldehyde before sectioning at -20 °C (Leica CM1950). The coronal brain tissue slices (20μm) were fixed with 4% paraformaldehyde at room temperature for 30 min then washed three times with a phosphate buffer solution (PBS) (Solarbio). The sections were then treated with 0.3% Triton X-100 (in PBS) for 20 min then washed three times with PBS. Subsequently, sections were blocked with a 1% bovine serum albumin (BSA) solution at room temperature for 1 hour. The obtained sections were then incubated overnight at 4°C with the corresponding primary antibody. Sections were incubated for 1 hour in the dark the next day with the corresponding secondary Alexa Fluor antibody after washing. The slides were then washed, mounted in the dark. A laser confocal microscope (Olympus, FV1000, Japan) was used for image acquisition.

**Transmission Electron Microscopy (TEM)**

After identification and dissection of the hippocampal DG region, tissues (1mm^3^) were fixed in 2.5% glutaraldehyde at 4°C. Tissues were then sectioned and stained (lead). Sections (at least 10 per subject) were finally selected and photographed by a transmission electron microscope (Hitachi H-7650, Japan) (40000×). The subsequent measurements were performed by Image-Pro Plus 6.0 (Media Cybernetics, USA).


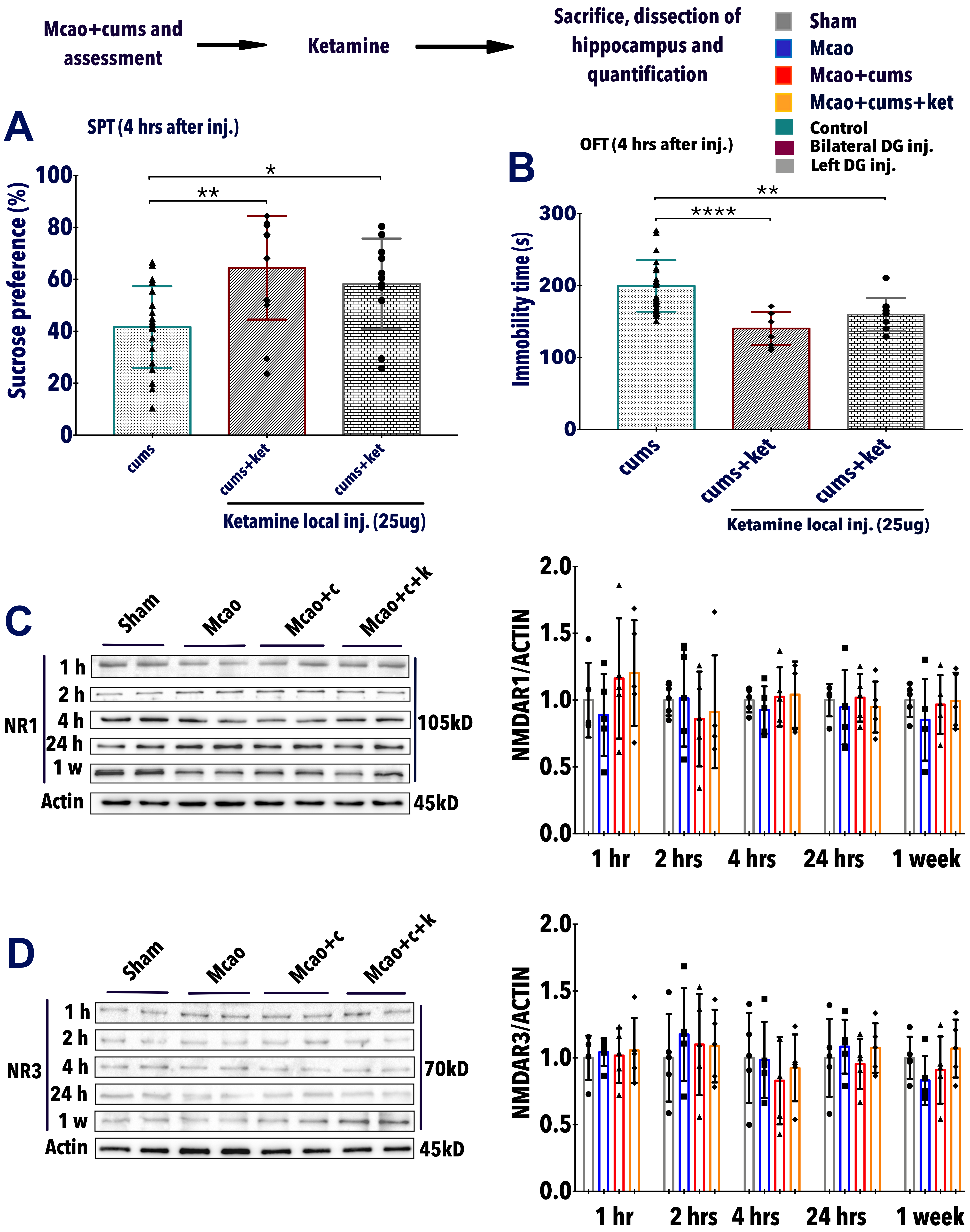


**Figure S1: Pre-study analysis of the effectiveness of drug delivery and effects of ketamine on remaining NMDAR subunits**

OFT, SPT, and western blotting were conducted to assess the viability of the chosen drug delivery method as well as the impact of ketamine on the remaining NMDAR subunits. (A) Pre-study comparison of anhedonia between the various delivery methods. (B) Pre-study immobility results between the two delivery options. (C) Representative immunoblots and protein levels of NMDAR1 (NR1) in the hippocampal DG region of various study groups. (D) Representative immunoblots and protein levels of **NMDAR3** (NR3) in the hippocampal DG region of each subjects. 1 hr = 1 hour, 2 hrs = 2 hours, 4 hrs = 4 hours, 24 hrs = 24 hours, and 1w = 1 week. Inj. = injection; DG = dentate gyrus, Mcao+c = Mcao+cums; Mcao+c+k =Mcao+cums+ketamine. Statistical significance was determined by one-way ANOVA followed by Tukey’s multiple comparison tests. The values are expressed as the mean ± standard deviation. n = 6 per group for western blot results and n = 12 for OFT and SPT. ∗ *p <* 0 05, ∗∗ *p <* 0 005, ∗∗∗ *p <* 0 0005, ∗∗∗∗ *p <* 0 0001.

**
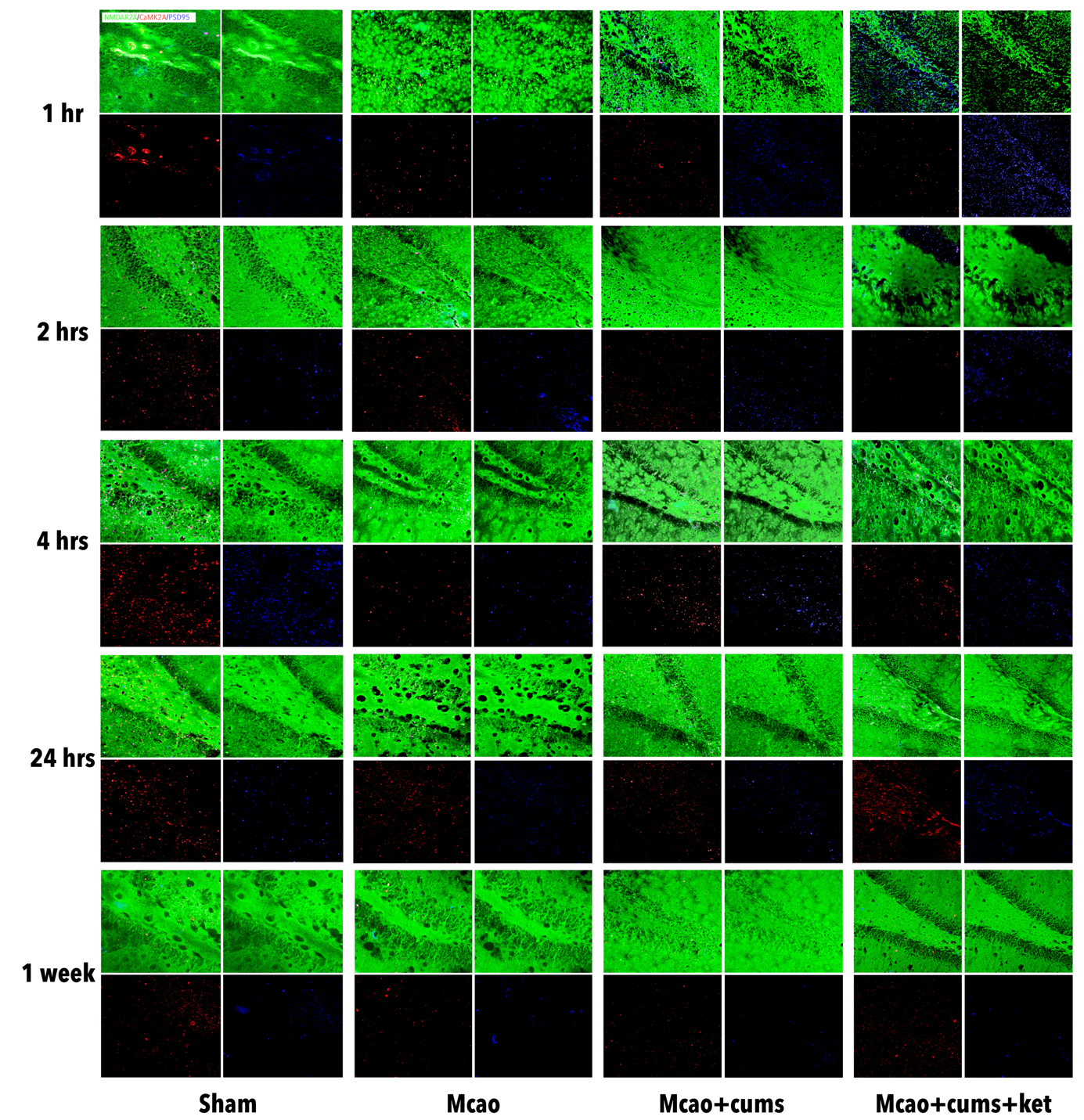
**

**Figure S2: Effects of ketamine administration on the expressions of NMDAR2A, CaMK2A, and PSD95 in various groups**

Representative immunofluorescent photomicrographs (200x) of NMDAR2A/CaMK2A/PSD95 (green/red/blue) in the hippocampal DG region of depressive MCAO rats were acquired 1 hour, 2 hours, 4 hours, one day, and one week after ketamine administration. Scale bar: 100μm. n = 6 per group. 1 hr = 1 hour, 2 hrs = 2 hours, 4 hrs = 4 hours, and 24 hrs = 24 hours.


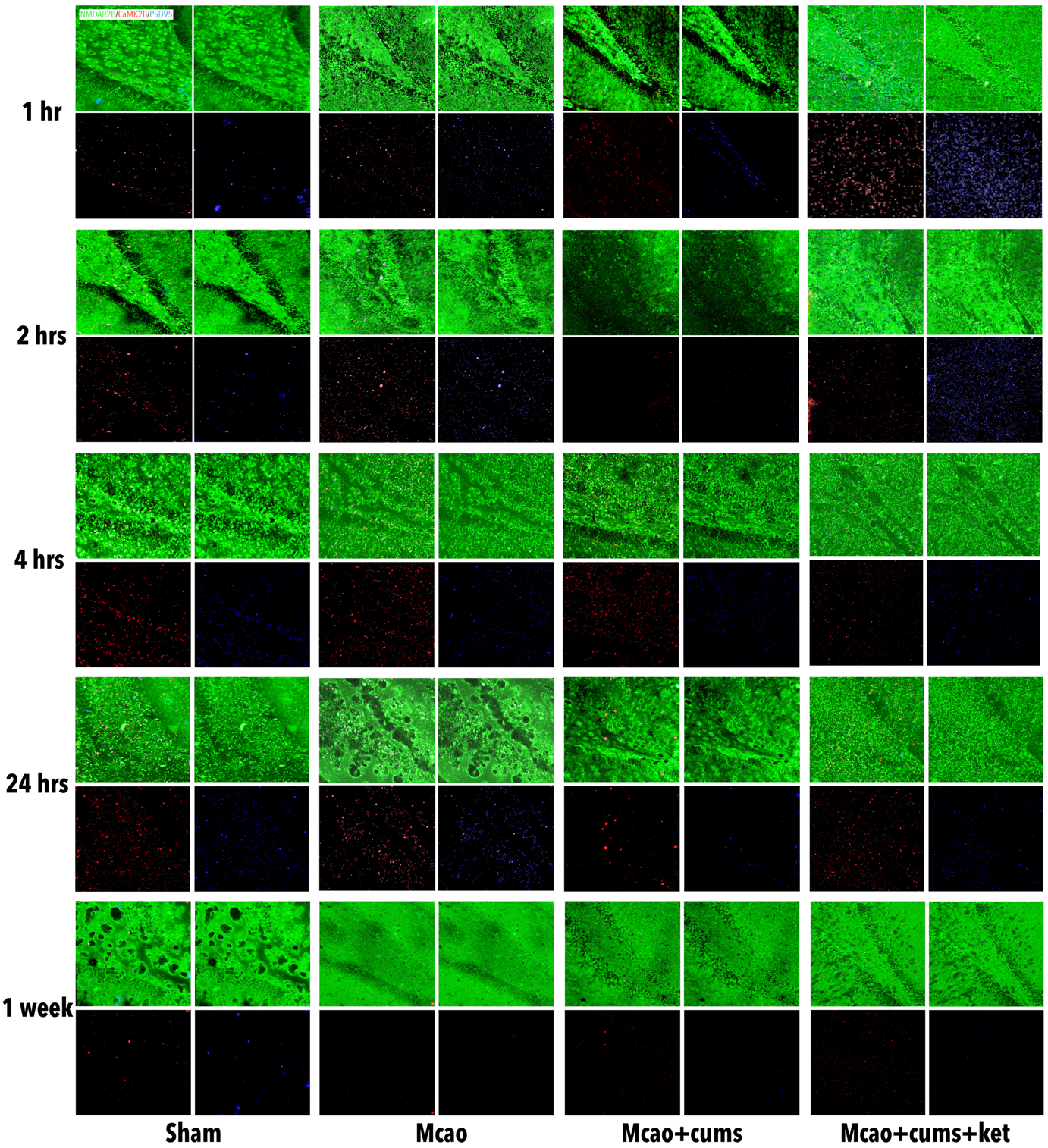


**Figure S3: Effects of ketamine administration on the expressions of NMDAR2B and CaMK2B in various groups**

Representative immunofluorescent photomicrographs (200x) of NMDAR2B/CaMK2B/PSD95 (green/red/blue) in the hippocampal DG region of depressive MCAO rats were pictured 1 hour, 2 hours, 4 hours, one day, and one week after ketamine administration. Scale bar: 100μm. n = 6 per group. 1 hr = 1 hour, 2 hrs = 2 hours, 4 hrs = 4 hours, and 24 hrs = 24 hours.


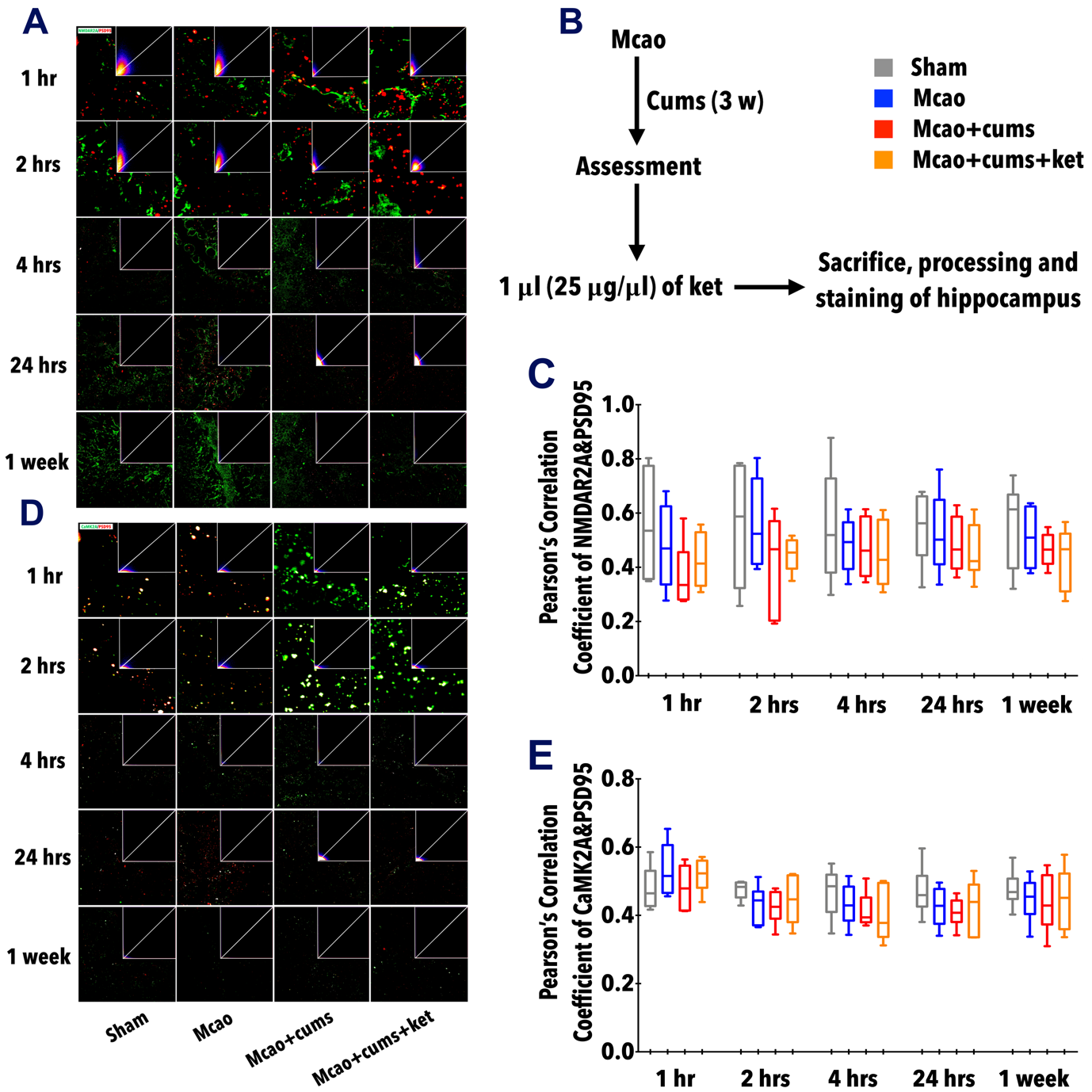


**Figure S4: Analysis of the relationship between components of the NMDAR/CaMKII pathway and changes at the postsynaptic level by co-localization**

(A) Representative confocal images of the relationship between NMDAR2A (labeled in green) and PSD95 (labeled in red). The upper right corner of each image shows the corresponding scattergram with pixel concentration along the diagonal, depending on the degree of co-localization. (B) Procedures before image acquisition. (C) Co-localization between NMDAR2A and PSD95, as analyzed by Pearson’s correlation coefficients. (D) Representative confocal images of the relationship between CaMK2A (labeled in green) and PSD95 (labeled in red). The upper right corner of each image shows the corresponding scattergram with pixel concentration along the diagonal, depending on the degree of co-localization. (E) Co-localization between CaMK2A and PSD95, as analyzed by Pearson’s correlation coefficients. Scale bar: 20μm. Pearson’s correlation coefficient analysis was based on ten sight fields in each group. n = 6 per group. 1 hr = 1 hour, 2 hrs = 2 hours, 4 hrs = 4 hours, 24 hrs = 24 hours, and 3 w = 3 weeks. ∗ *p <* 0 05, ∗∗ *p <* 0 005, ∗∗∗ *p <* 0 0005, ∗∗∗∗ *p <* 0 0001.

**Supplemental reference**

Longa, E. Z., P. R. Weinstein, S. Carlson and R. Cummins (1989). "Reversible middle cerebral artery occlusion without craniectomy in rats." Stroke **20**(1): 84-91.
